# Effectiveness of a novel gas‐release endorectal balloon in the removal of rectal gas for prostate proton radiation therapy

**DOI:** 10.1120/jacmp.v13i5.3945

**Published:** 2012-09-06

**Authors:** Landon S. Wootton, Rajat J. Kudchadker, A. Sam Beddar, Andrew K. Lee

**Affiliations:** ^1^ Department of Radiation Physics The University of Texas MD Anderson Cancer Center Houston TX; ^2^ Department of Radiation Oncology The University of Texas MD Anderson Cancer Center Houston TX

**Keywords:** endorectal balloon, prostate, rectal gas, radiation therapy

## Abstract

Endorectal balloons (ERBs) are routinely used in prostate proton radiation therapy to immobilize the prostate and spare the rectal wall. Rectal gas can distend the rectum and displace the prostate even in the presence of ERBs. The purpose of this work was to quantify the effects an ERB with a passive gas release conduit had on the incidence of rectal gas. Fifteen patients who were treated with a standard ERB and 15 with a gas‐release ERB were selected for this retrospective study. Location and cross‐sectional area of gas pockets and the fraction of time they occurred on 1133 lateral kilovoltage (kV) images were analyzed. Gas locations were classified as trapped between the ERB and anterior rectal wall, between the ERB and posterior rectal wall, or superior to the ERB. For patients using the standard ERB, gas was found in at least one region in 45.8% of fractions. Gas was trapped in the anterior region in 37.1% of fractions, in the posterior region in 5.0% of fractions, and in the sigmoid region in 9.6% of fractions. For patients using the ERB with the gas‐release conduit, gas was found in at least one region in 19.7% of fractions. Gas was trapped in the anterior region in 5.6% of fractions, in the posterior region in 8.3% of fractions, and in the sigmoid region in 7.4% of fractions. Both the number of fractions with gas in the anterior region and the number of fractions with gas in at least one region were significantly higher in the former group than in the latter. The cross‐sectional area of trapped gas did not differ between the two groups. Thus gas‐release balloon can effectively release gas, and may be able to improve clinical workflow by reducing the need for catheterization.

PACS number: 87.56.Da

## I. INTRODUCTION

As radiation therapy technology has matured, the delivery of increasingly conformal radiation fields with commensurately smaller margins at higher doses has become commonplace. This approach to treating prostate cancer has been shown to achieve better tumor control^1^, ^2^ with less radiation damage to critical organs and a subsequent decrease in the incidence of toxic effects.^3^, ^6^ However, the high conformality, elevated dose, and small margins also make this approach more vulnerable to uncertainties that displace the target from the treatment field, such as interfractional and intrafractional organ movement and setup errors.^(7)^

In the treatment of prostate cancer, numerous methods have been developed to combat these uncertainties. These methods include daily imaging of implanted fiducials to reduce setup uncertainty,^8^, ^9^ endorectal balloon (ERB) to reduce prostate movement and provide more reproducible setup by displacing rectal contents that shift the prostate,^10^, ^12^ and injecting saline into the rectum to displace rectal gas and to increase the homogeneity of the density of materials in the beam path.^(13)^

**Figure 1:**
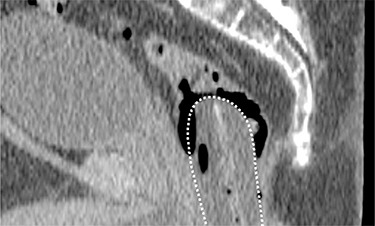
Sagittal CT image of a patient with a large amount of rectal gas. The rectal balloon is delineated by the white dotted line. Gas is trapped and has accumulated between the endorectal balloon and the rectal wall, distending the rectum and possibly displacing the prostate. Air bubbles are also present within the rectal balloon.

Rectal gas is a consistent problem in prostate cancer radiation therapy. Gas distends the rectum and thereby displaces the prostate (Fig. 1). Furthermore, the presence of gas is neither predictable nor reproducible, leading to unpredictable prostate movement between treatments and during the delivery of treatment.^(14)^ This effect is especially influential in proton therapy, for which the presence of rectal gas can significantly alter the dose distribution; protons are highly sensitive to the medium they travel through, and when tissue is displaced by gas, protons penetrate further than planned. This increased proton penetration results in unnecessary irradiation of normal tissue.

ERBs are commonly used as a means of reducing prostate movement. Prior to simulation and treatment, a balloon is inserted into the patient's rectum and then inflated. Typically water filled balloons are utilized in patients receiving proton radiation therapy, while air filled balloons are utilized in patients receiving photon or X‐ray therapy. The introduction of the balloon helps prevent posterior prostate motion by immobilizing the prostate against the pubic symphysis in the anterior direction and against the full bladder in the superior direction. In addition to confining the location of the prostate, the rectal balloon also displaces the rectum's contents, thus decreasing variability in prostate position due to gas or stool.^(15)^ However, rectal gas that accumulates superior to the balloon, or that is trapped between the balloon and the rectal wall, can still displace the prostate and may require removal if present in significant amounts.^10^, ^16^ Gas can potentially be removed by inserting a catheter before the balloon is inserted for daily radiation treatments. Another possibility for eliminating rectal gas is a passive gas‐release ERB.

Recent attempts to reduce the effects of rectal gas have included using ERBs designed to passively alleviate rectal gas. However, the effectiveness of these gas‐release ERBs has yet to be assessed. The purpose of this study was to quantitatively evaluate the effect of the new gas‐release balloon on the amounts and locations of rectal gas during proton treatment for prostate cancer.

## II. MATERIALS AND METHODS

Thirty prostate cancer patients treated with proton therapy in 2011 at our institution were included in this institutional review board‐approved retrospective study. The average age of the patients at the time of treatment was 62 years old (range: 46–72). All patients were treated for T1c, T2c, or T2a prostate cancer. Parallel opposed passively scattered proton beams were used to treat each patient to 76 Gy (RBE) in 38 fractions (2 Gy/fx).

**Figure 2:**
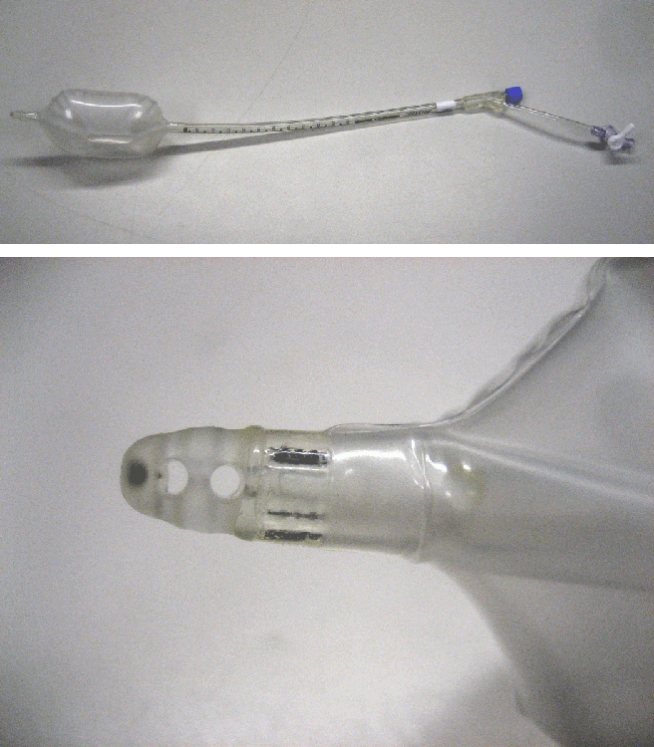
Radiadyne Gas‐Release Balloon (top) and a close‐up of the opening of the gas release conduit at the tip of the balloon (bottom). Gas travels into the tip, through the stem of the balloon and out of the stem just below the point at which the stem bifurcates. The long half of the bifurcated stem is used for inflating and deflating the balloon.

The new ERB design (Radiadyne, Houston, TX) incorporates an open conduit through the balloon stem (Fig. 2). The purpose of the conduit is to release rectal gas while the balloon is being inserted so when the balloon is inflated, no gas will be trapped between the balloon and the rectal wall. After inflation, the gas‐release conduit continues to provide an escape path for any additional gas arriving at the superior end of the balloon, thus preventing gas accumulation. The balloon conduit is always open and does not require any action to function.

Fifteen of the selected patients had been treated using a standard ERB (RB‐100; Radiadyne, Houston, TX) and the other 15 using the gas‐release ERB (GRB‐90F; Radiadyne). The gas‐release ERB incorporates a passive gas‐release conduit through the balloon stem, a fiducial bead at the balloon tip, and a stopper at the balloon base to facilitate reproducible depth of insertion. The ERBs were inserted immediately before simulation computed tomography (CT) and all treatment fractions. All balloons were inflated with 100 mL of water so that their density would be homogeneous with that of the surrounding tissue.

We analyzed the data from the lateral kV images that were taken at the time of daily treatment for patient alignment and assessment of gas in the rectum. The distance from the X‐ray source to the imager (SID) was 323 cm. The distance from the X‐ray source to the gantry isocenter (SAD) was 273 cm. In the resulting images, a pixel corresponded to a 0.3920 by $0.3920\;{\text{mm}}^{2}$ area on the plane containing the isocenter. A total of 1133 images (36–38 per patient) were available for analysis. Patient information was removed from each image. Information identifying the model of the ERB used was also removed to avoid biasing the study. This involved obscuring regions of images in which the presence or absence of landmarks (such as the fiducial at the tip of the gas‐release ERB or the stopper at its base) would identify the model of ERB used.

**Figure 3:**
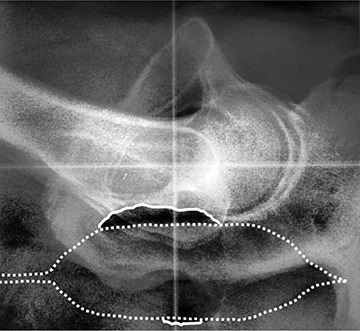
Sample daily kV image of a patient in our study showing the rectal balloon (dotted white line) and anterior and posterior gas bubbles (solid white lines).

After preparing the images, we inspected each image for the presence of gas. Pockets of gas were identified according to several characteristics. First, pockets of rectal gas typically have well‐delineated borders. Second, gas pockets cause distinct changes between daily images of the same patient; by examining multiple images in succession, gas pockets can be distinguished from anatomical features that resemble gas pockets. Finally, before an image feature is identified as gas, the possibility that it is an air bubble in the ERB has to be ruled out. Because patients are imaged while supine, bubbles typically float anteriorly; thus, bubbles in the ERB can be identified by their location in the anterior region of the rectum, their curved anterior border matching the contour of the balloon, and their level posterior border. Figure 3 displays a daily kV image with the rectal balloon with two gas pockets identified.

After identifying the gas pockets in all images, we recorded the location and cross‐sectional area of each pocket. The location of each gas pocket was classified as occurring in one of three regions: between the ERB and the anterior rectal wall, between the ERB and the posterior rectal wall, and superior to the ERB in the sigmoid colon (limited to 7 cm superior from the top of the ERB and 4 cm anterior or posterior to it). The cross‐sectional areas of the gas pockets were also recorded. The cross‐sectional area of the gas pockets was determined by measuring the dimensions of each pocket in terms of pixels and converting to physical distance using a conversion of 0.3920 mm per pixel. This method assumes that gas pockets lie in the plane containing the isocenter. This assumption is valid as the rectum is directly posterior to the prostate where the isocenter is placed. The number, size, and location of gas pockets were recorded for each fraction for each patient.

The percentage of fractions with rectal gas in at least one region was calculated for each patient, as were the percentages of fractions with gas in the anterior, posterior, and sigmoid regions. The mean cross‐sectional area of rectal gas pockets was also calculated for each region for each patient. These calculated values were then averaged over the standard ERB and gas‐release ERB patient groups. Finally, a two‐tailed t‐test for independent samples was used to determine whether the differences between the two groups' results were statistically significant. Statistical analysis was performed in Microsoft Excel 2007 and p‐values less than 0.05 were considered to indicate a statistically significant difference.

## III. RESULTS

**Figure 4:**
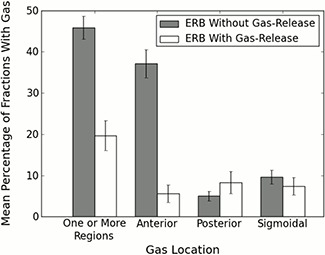
Mean frequency of rectal gas according to region and type of ERB used. The mean incidences of gas in one or more regions and in the anterior region differed at statistically significant levels (*p* < 0.00001 and *p* < 0.0000001, respectively) between the standard and gas‐release ERBs. Error bars represent the standard deviation of the mean of within‐patient means.

For the patients using the standard ERB (i.e., an ERB with no gas‐release mechanism), gas was present in one or more of the three regions in 45.8% ±2.8% of fractions (mean ± standard deviation; Fig. 4). Gas was present in the anterior region in 37.1%±3.4% of fractions, in the posterior region in 5.0%±1.2%, and in the sigmoid region in 9.6%±1.7%. For patients using the gas‐release ERB, gas was present in one or more regions in 19.7%±3.6% of fractions (anterior region, 5.6%±2.1% of fractions; posterior region, 8.3%±2.7% of fractions; and sigmoid region, 7.4%±2.1% of fractions). A comparison between the two patient groups indicates that the difference between the numbers of fractions with gas in one or more regions was statistically significant (p<0.00001), as was the difference in the number of fractions with gas in the anterior region (p<0.0000001). The differences between the number of fractions with gas in the posterior and sigmoid region were not statistically significant.

**Figure 5:**
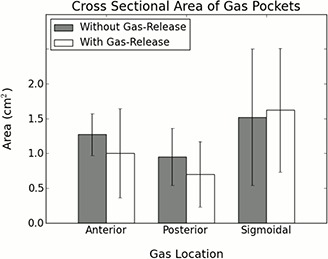
Mean cross‐sectional area of rectal gas pockets by location. Error bars represent the standard deviation of the mean of within‐patient means.

For patients treated using the standard ERB, the mean cross‐sectional areas of gas pockets in the anterior, posterior, and sigmoid regions were $1.3\;{\text{cm}}^{2}\pm 0.3\;{\text{cm}}^{2}$, $1.0\;{\text{cm}}^{2}\pm 0.4\;{\text{cm}}^{2}$, and $1.5\;{\text{cm}}^{2}\pm 1.0\;{\text{cm}}^{2}$, respectively (Fig. 5). For patients with the gas‐release ERB, the mean cross‐sectional areas of gas pockets in the anterior, posterior, and sigmoid regions were $1.0\;{\text{cm}}^{2}\pm 0.6\;{\text{cm}}^{2}$, $0.7\;{\text{cm}}^{2}\pm 0.5\;{\text{cm}}^{2}$, and $1.6\;{\text{cm}}^{2}\pm 0.9\;{\text{cm}}^{2}$, respectively. Differences between the two groups were not significant. These results are presented in Fig. 5.

## IV. DISCUSSION

Our analysis indicates that the gas‐release ERB significantly decreased the number of fractions in which gas was present, primarily by decreasing the incidence of gas trapped between the rectal balloon and the anterior rectal wall. Therefore, we recommend that gas‐release ERBs be used in patients undergoing radiation therapy for prostate cancer. The prevalence of gas that we found in the anterior region is consistent with previous findings.^(17)^ Knowing that gas is most likely to be trapped in the anterior region is important because gas trapped there will not only displace the prostate, but also push the anterior rectal wall into the treatment field. This could potentially alter the prostate and rectal dose distribution and possibly the treatment outcome. In X‐ray conformal or intensity‐modulated radiation therapy, such a change in dose distribution would likely be small, and the negative effects of gas would stem mostly from organ displacement.^(18)^ However, in proton radiation therapy, gas in the treatment field can escalate dose to normal tissue to an unacceptably high level because of the extreme sensitivity of protons to the medium they travel through. A proton beam's range, and thus energy deposition, is extremely sensitive to the density of the medium through which the beam passes. For example, Lin et al.^(19)^ found that anterior gas pockets were capable of causing excessively high dose to small regions of the femoral heads when patients were treated with lateral oblique proton beams. Gas in the posterior and sigmoid regions can also displace the prostate, but this occurrence was not common when using either model of ERB in our analysis.

The cross‐sectional areas of gas pockets did not change significantly with respect to ERB model used demonstrating that, although gas occurs less often with the gas‐release balloon, the severity of the gas is not decreased by it. Thus, catheterization or some other gas removal technique will continue to be necessary. A possible explanation for this result could be hasty gas‐release ERB insertion that does not allow time for gas to escape through the conduit before being trapped when the balloon is inflated. If this is the case, the gas‐release ERB could be used to better advantage by slow, careful insertion; however, this possibility remains to be tested.

An important advantage of passive gas release over catheterization is that it continues to work during patient treatment. Although existing rectal gas may be removed by a catheter at the beginning of treatment, gas may continue to build up during the course of the treatment. This possibility was documented by Ogino et al.,^(14)^ who monitored patients with cine magnetic resonance imaging for rectal contents and prostate displacement over the course of eight minutes. Patient rectums accumulated gas during that period even though a rectal gas removal technique was administered just prior to observation, and this accumulation resulted in prostate displacements of up to 6 mm. A passive gas‐release system on the ERB provides an escape for rectal gas, preventing it from accumulating and reaching the balloon during treatment. Thus, a gas‐release ERB would be expected to show advantages even greater than those presented here if the entire course of treatment were taken into account. Future studies using cine magnetic resonance imaging during treatment or additional imaging at the end of treatment are recommended.

This study was subject to a few limitations. All patients received kV imaging daily, but only one or two CT scans over the entire treatment course. Thus, to obtain sufficient amount of data to determine how often rectal gas occurs with reasonable statistical certainty, we were limited to analyzing daily kV images. However, kV images lack the volumetric information of CT images, and therefore, only the cross‐sectional areas of gas pockets could be compared, rather than more robust measures such as volumetric data. Another limitation stems from our decision not to analyze both anterior–posterior and lateral images together. To do so would have required examination of twice as many images and would have required that pockets visible on both images be identified to avoid counting them as separate pockets. Because of the large number of fractions for which we wished to obtain data (more than 1000), we chose to use only lateral images. These images provided critical information on rectal gas in the anterior rectal region that was in the treatment field, whereas anterior–posterior kV images, which lack depth information, would not have allowed us to distinguish between gas trapped anteriorly and posteriorly.

## V. CONCLUSIONS

Lateral kV images from daily radiation treatments of 30 prostate cancer patients treated at our institution were examined for the presence of rectal gas. A standard ERB was used for 15 patients, and a modified ERB designed to passively allow gas release was used for the remaining 15 patients. The modified ERB significantly decreased the overall frequency of fractions with gas present in any region by decreasing the frequency of fractions with gas present between the rectal balloon and the anterior rectal wall, the most common location of rectal gas. We conclude, therefore, that the gas‐release ERB effectively removes rectal gas and should be used in patients receiving proton radiation therapy.

## References

[1] Pollack A , Zagars GK , Starkschall G , et al. Prostate cancer radiation dose response: results of the M.D. Anderson phase III randomized trial. Int J Radiat Oncol Biol Phys. 2001;53(5):1097–105. 10.1016/s0360-3016(02)02829-8 12128107

[2] Hanks GE , Hanlon AL , Epstein B , Horwitz EM . Dose response in prostate cancer with 8–12 years' follow‐up. Int J Radiat Oncol Biol Phys. 2002;54(2):427–35. 12243818 10.1016/s0360-3016(02)02954-1

[3] Zelefsky MJ , Cowen D , Fuks Z , et al. Long term tolerance of high dose three‐dimensional conformal radiotherapy in patients with localized prostate carcinoma. Cancer. 1999;85(11):2460–68. 10357419 10.1002/(sici)1097-0142(19990601)85:11<2460::aid-cncr23>3.0.co;2-n

[4] Zelefsky MJ , Fuks Z , Leibel SA . Intensity‐modulated radiation therapy for prostate cancer. Semin Radiat Oncol. 2002;12(3):229–37. 12118388 10.1053/srao.2002.00000

[5] Storey MR , Pollack A , Zagars G , Smith L , Antolak J , Rosen I . Complications from radiotherapy dose escalation in prostate cancer: preliminary results of a randomized trial. Int J Radiat Oncol Biol Phys. 2000;48(3):635–42. 11020558 10.1016/s0360-3016(00)00700-8

[6] Teh BS , Mai W , Uhl BM , et al. Intensity‐modulated radiation therapy (IMRT) for prostate cancer with the use of a rectal balloon for prostate immobilization: acute toxicity and dose‐volume analysis. Int J Radiat Oncol Biol Phys. 2001;49(3):705–12. 11172952 10.1016/s0360-3016(00)01428-0

[7] Langen KM , Jones DTL . Organ motion and its management. Int J Radiat Oncol Biol Phys. 2001;50(1):265–78. 11316572 10.1016/s0360-3016(01)01453-5

[8] Chen J , Lee RJ , Handrahan D , Sause WT . Intensity‐modulated radiotherapy using implanted fiducial markers with daily portal imaging: assessment of prostate organ motion. Int J Radiat Oncol Biol Phys. 2007;68(3):912–19. 17459605 10.1016/j.ijrobp.2007.02.024

[9] Schallenkamp JM , Herman MG , Kruse JJ , Pisansky TM . Prostate position relative to pelvic bony anatomy based on intraprostatic gold markers and electronic portal imaging. Int J Radiat Oncol Biol Phys. 2005;63(3):800–11. 16199313 10.1016/j.ijrobp.2005.02.022

[10] Smeenk RJ , Teh BS , Butler EB , van Lin EN , Kaanders JH . Is there a role for endorectal balloons in prostate radiotherapy? A systematic review. Radiother Oncol. 2010;95(3):277–82. 20451274 10.1016/j.radonc.2010.04.016

[11] D'Amico AV , Manola J , Loffredo M , et al. A practical method to achieve prostate gland immobilization and target verification for daily treatment. Int J Radiat Oncol Biol Phys. 2001;51(5):1431–36. 11728704 10.1016/s0360-3016(01)02663-3

[12] McGary JE , Teh BS , Butler B , Grant W . Prostate immobilization using a rectal balloon. J Appl Clin Med Phys. 2002;3(1):6–11. 11817999 10.1120/jacmp.v3i1.2590 PMC5724550

[13] Vargas C , Mahajan C , Fryer A , et al. Rectal dose‐volume differences using proton radiotherapy and a rectal balloon or water alone for the treatment of prostate cancer. Int J Radiat Oncol Biol Phys. 2007;69(4):1110–16. 17967305 10.1016/j.ijrobp.2007.04.075

[14] Ogino I , Kaneko T , Suzuki R , et al. Rectal content and intrafractional prostate gland motion assessed by magnetic resonance imaging. J Radiat Res. 2011;52(2):199–207. 21436610 10.1269/jrr.10126

[15] van Lin EN , Hoffman AL , van Kollenburg P , Leer JW , Visser AG . Rectal wall sparing effects of three different endorectal balloons in 3D conformal and IMRT prostate radiotherapy. Int J Radiat Oncol Biol Phys. 2005;63(2):565–76. 16168848 10.1016/j.ijrobp.2005.05.010

[16] Cho JH , Lee CG , Kang DR , et al. Positional reproducibility and effects of a rectal balloon in prostate cancer radiotherapy. J Korean Med Sci. 2009;24(5):894–903. 19794990 10.3346/jkms.2009.24.5.894 PMC2752775

[17] Stroom JC , Kroonwijk M , Pasma KL , Koper PCM , van Dieren EB , Heijmen BJM . Detection of internal organ movement in prostate cancer patients using portal images. Med Phys. 2000;27(3):452–61. 10757597 10.1118/1.598913

[18] Ebert MA and Spry NA . Dose perturbation by air cavities in megavoltage photon beams: Implication for cavity surface doses. Australas Radiol. 2001;45(2):205–10. 11380365 10.1046/j.1440-1673.2001.00900.x

[19] Lin L , Vargas C , Hsi W , et al. Dosimetric uncertainty in prostate cancer proton radiotherapy. Med Phys. 2008;35(11):4800–07. 19070212 10.1118/1.2982242

